# Cholera and the Water Supply in the South Districts of London in 1854

**Published:** 1856-10

**Authors:** John Snow


### CHOLERA AND THE WATER SUPPLY IN THE SOUTH DISTRICTS OF LONDON, IN 1854.

By JOHN SNOW, M.D.

In the summer of 1849, I published certain conclusions at which I have arrived with regard to Asiatic cholera, and the facts and reasonings which had led to them. The following is a very brief outline of these views. The cholera commences as an affection of the alimentary canal, and not with general illness; there is no evidence of poisoning of the blood in this disease, except in some cases where secondary fever occurs; there is conclusive evidence that cholera may be communicated from person to person, and it follows, therefore, that the morbid matter which produces the disease is applied to the interior of the alimentary canal, where it increases and multiplies during the period of so-called incubation, and passes off, during the attack, to cause fresh cases when suitable opportunities occur. Various circumstances connected with the propagation of cholera seemed in accordance with the above view of its pathology. Thus, it was observed to pass frequently from person to person in the crowded habitations of the poor, who eat, drink, cook, and sleep in the same apartment, and pay little or no regard to cleanliness, who live, in fact, under circumstances where the sudden and copious evacuations of cholera, soiling the bed and body linen, would not fail to contaminate the hands of the patient and his attendants, and be thence transferred to any food they might touch. The absence of colour and odour in the evacuations could not help to favour this result. The social visitor who came to see the poor patient, or attend his funeral, frequently suffered, whilst the medical man, and others who partook of no food in the apartment, and who washed their hands when requisite, escaped. The mining districts of this country have suffered excessively from cholera in each epidemic, an event which might be explained by the following circumstances when taken in connexion with the above view of the cause of the disease. The miners stay eight or nine hours at a time in the pits, and take food with them, which they eat invariably with unwashed hands, and without knife and fork, whilst the pits are without privies, and are generally extremely foul and dirty. The entire absence of daylight must also cause the workmen to take much more dirt with their food than they aware of. It occurred to me, as soon as I began to entertain the above opinions, that if the cholera excreta could reproduce the disease in the way just mentioned, they might also do so when diffused in water taken as drink, and that unless this were the case, the whole of the phenomena of cholera, as an epidemic, could not be explained. I, therefore, sought anxiously, and waited patiently, for some confirmation of this part of the subject before I should make my views known. Two outbreaks of cholera occurred, however, about the end of July 1849, one in Horsleydown, and the other in the Wandsworth Road, which I investigated, and which afforded what I considered conclusive evidence on the subject. The water drank by the persons attacked in each of these outbreaks had received, amongst other impurities, what must have come from a patient previously ill of the disease. I was able also to point out that the cholera was prevailing most in those districts of the metropolis which received their supply of water from certain parts of the Thames which contained the sewage of the town, and, consequently, whatever proceeded from the cholera patients. Before the end of 1849 I was able to show that a very close connexion existed between the mortality from cholera and the nature of the water supply, not only in London, but throughout the country. This connexion was very evident in certain towns, as Exeter and Hull, where the supply of water had been changed between the epidemic of 1832 and that of 1849. Where a polluted supply was changed for an unpolluted one, the cholera was almost prevented; and where a scanty but unpolluted supply had been changed for one contaminated with the sewage of the town, the epidemic prevailed to a fearful extent. The attention of Dr. Wm. Budd and Dr. Farr was directed to this subject, with the result of confirming what I had stated.

Between the epidemics of 1849 and that of 1853, one of the water companies supplying the south districts of London changed its source of supply from the middle of the town, near the foot of the Hungerford Suspension Bridge, to Thames Ditton, at a part of the river which is beyond the influence of the tide, and, therefore, out of reach of the sewage of the metropolis. In the autumn of 1853 it was shown by Dr. Farr* that the districts partly supplied by this, the Lambeth Water Company, with improved water, suffered less than the districts supplied entirely by the Southwark and Vauxhall Company with the water from the river at Battersea Fields, although in 1849 they had suffered rather

---

* Weekly Returns of Deaths, November.

more than the latter districts. By showing the water supply in subdistricts, and thus getting a more correct line of demarcation, I was able to point out* that the advantage in favour of the population partly supplied with the purer water was even greater than Dr. Farr had indicated.

I had learnt from the evidence of Mr. Quick in the *Health of Towns Reports*, that the division of the houses, between the Lambeth Company on the one hand, and the Southwark and Vauxhall Company on the other, was not such as obtains in the north districts of London, where a parish is often divided between two water companies, but where one company always leaves off at the point at which the other begins. Throughout the greater part of Lambeth and Southwark, the whole of Newington, and a part of Camberwell, however, the supply of the two companies above mentioned is actually intermixed, the pipes of both companies going down the same streets, in consequence of the active competition which once existed between three water companies, two of which have since amalgamated and come to an agreement with the other—the Lambeth company. Observing, therefore, when the cholera returned in 1854, that there was the same advantage in favour of the districts partly supplied with water from Thames Ditton, I determined to make an inquiry, the idea of which I had previously entertained. It was obvious that, if the diminished mortality depended on the improved supply of water, the benefit of the whole diminution would be enjoyed by the inhabitants of houses having this supply, whilst the population receiving impure water would suffer as much as that of the districts which received the same water, and no other. This point could be determined by ascertaining the water supply of every house in which a fatal attack of cholera might occur. After commencing the inquiry I found that the circumstances were calculated for affording even more conclusive evidence than I had anticipated. The pipes of the two water companies not only passed down all the streets, but into nearly all the courts and alleys. A single house often had a different supply from that on either side. Each water company supplied alike both rich and poor, and thus there was a population of 300,000 persons, of various conditions and occupations, intimately mixed together, and divided into two groups by no other circumstance than the difference of water supply. One group supplied with water contaminated, to a large extent,

* On the Mode of Communication of Cholera, 2nd edit., p. 73.

with the sewage of London, and the other receiving a supply altogether free from such impurity.

I took great care to ascertain the nature of the water supply correctly in every instance. I did not rest content with the mere reply of the resident, or the appearance of the water, without other evidence, such as the production of the receipt for the water rate. I was also assisted very much by the application of a chemical test to the water, for throughout all the dry weather, which lasted whilst my inquiries were being made, a mixture of sea water extended further up the Thames than usual, and the water of the Southwark and Vauxhall Company contained nearly forty grains of common salt per gallon, whilst that of the Lambeth Company contained only ·95 of a grain. These analyses were verified in numerous cases where the source of the water could be proved clearly by other evidence. For the first four weeks of the epidemic I employed the list of deaths from cholera published in the Weekly Returns of the Registrar-General, and for the next three weeks, during which my inquiry extended, I was kindly permitted to copy the addresses of persons dying of cholera at the General Register Office. My personal inquiry extended over every subdistrict to which the supply of the Lambeth Water Company extended, and it, therefore, included all the area in which the supply of the two companies was intermixed in the manner explained above.

At the time I was making my inquiry, the entire number of houses supplied by each water company was known, from a return made to Parliament, but the number of houses supplied in each district and subdistrict by each company respectively was not known. In order, therefore, to see the exact bearing of my results, I found it desirable to extend the inquiry over the districts supplied exclusively by the Southwark and Vauxhall Company; for this purpose I obtained the assistance of Mr. Whiting, a medical man, who took great pains with his part of the inquiry, which was merely to ascertain whether the houses in which fatal attacks had taken place were supplied by the Southwark Company, or from some other source, as a pump well or tidal ditch. His inquiry extended over the first four weeks of the epidemic.

I gave a copy of the first results of my inquiry to Dr. Farr, to whom I was indebted for facilities very kindly afforded: and Dr. Farr being much struck with these results, instituted a continuance of the inquiry through the district registrars, who were requested to make a return of the supply of water to each house in which a fatal attack of cholera might occur in all the south districts of London. As the registrars could not be expected to make a chemical analysis of the water, or to seek out the landlord or agent in cases where the tenant was not acquainted with the water supply, the question remained unanswered in a considerable number of instances, but the return was obtained for more than three-fourths of the deaths, and shows, no doubt, the correct proportion. Dr. Farr's inquiry commenced from the 27th of August, and extended to the close of the epidemic; and as my inquiry extended to August 26th, the water supply was obtained for the whole epidemic of 1854. It was only necessary to make a computation of the small number of attacks occurring in houses supplied by pump wells or some other source, in the three weeks—the 5th to the 7th inclusive—of the epidemic, in Bermondsey and the other districts which do not receive the Lambeth water. This computation was made according to the result ascertained in the previous four weeks, and must approach very nearly to the truth.

In treating of the general results of this inquiry, it is desirable to divide the epidemic into different periods, as the influence of the water supply was found to diminish in relative intensity as the epidemic progressed. In the first four weeks of the epidemic of 1854, that is, from July 9th to August 5th inclusive, there were 334 deaths from cholera in the districts to which the supply of the two water companies we are considering extends. The water supply in every one of these instances was made a matter of personal inquiry, and the result of each case was published by me in detail in the Appendix to a work on Cholera. In 286 instances the supply of the house in which the attack took place was that of the Southwark and Vauxhall Company; in 14 instances it was that of the Lambeth Company; in 4 cases the supply was from a pump well; in 26 cases the water was drawn direct from the river, or a canal, or a tidal ditch; and in 4 cases the supply could not be ascertained, owing to the address of the deceased persons, prior to the fatal attack, not being known. The number of houses supplied by the Southwark and Vauxhall Company was 40,046, having a population estimated by the Registrar-General* at 266,516, and the number of houses supplied by the Lambeth Company

* Weekly Returns for 1854, p. 433.

was 26,107, with an estimated population of 173,748; the mortality from cholera was, therefore, at the rate of 107 to each 100,000 inhabitants supplied by the former company, and 8 to each 100,000 supplied by the latter; in other words, the disease was between thirteen and fourteen times as fatal to the population having the impure water as to that having the improved supply. It is particularly worthy of remark that, during the four weeks of the epidemic we are now considering, there were but 563 deaths from cholera in the whole metropolis, of which 286, or more than one-half, occurred amongst the customers of the Southwark and Vauxhall Company, who comprise a little more than one-tenth of London, and a considerable number of the remaining deaths took place amongst mariners, and others employed amongst the shipping, who almost invariably draw their drinking water directly from the river; it is, therefore, evident that at this early period of the epidemic the impure water of the Thames was almost the exclusive means of the propagation of the malady.

In the next three weeks of the epidemic there were 1,180 deaths from cholera in the districts supplied by the two water companies. Of these, the fatal attack took place in 977 cases in houses supplied by the Southwark and Vauxhall Company; in 84 cases in houses supplied by the Lambeth company; in 101 instances the supply was from some other source; and in 18 cases it could not be ascertained, for reasons previously stated. Taking into account the population supplied respectively by each company, the mortality was, at this period of the epidemic, nearly eight times as great in that supplied by the Southwark and Vauxhall Company as in that supplied by the Lambeth Company.

During the last ten weeks of the epidemic, from August 27th to November 4th inclusive, 3,564 deaths occurred in the districts to which the supply of the two water companies extends, and the returns of the district registrars showed that in 2,443 cases the water supply of the house in which the fatal attack took place was that of the Southwark and Vauxhall Company; in 313 cases it was that of the Lambeth Company; in 207 instances the supply was from pump wells and other sources independent of the two water companies, and in 601 instances the supply was not ascertained.* These numbers show a mortality of 916 to each 100,000 inhabitants supplied by the Southwark and Vauxhall Company,

* Weekly Returns for 1854, pp. 514-18.

and 180 to each 100,000 supplied by the Lambeth Company; consequently, at this period of the epidemic, the mortality was still more than five times as great amongst the population supplied by the former company as amongst that supplied by the latter.

The results of my inquiry into the supply of water were, of course, obtained separately for each district and subdistrict in which the inquiry was made, and were so published; but I was unable at the time to show the relation between the supply of houses in which fatal attacks took place, and the entire supply of each district and subdistrict, on account of the latter circumstance not being known. I expressed myself as follows in an article which I published soon after my inquiry was made: "I hope shortly to learn the number of houses in each subdistrict supplied by each of the water companies respectively, when the effect of the impure water in propagating cholera will be shown in a very striking manner, and with great detail."* This information did not, however, come within my reach till recently, and not even then with all the accuracy I could desire. In the Report on the Cholera Epidemics of London as affected by the Consumption of Impure Water, lately written by Mr. Simon, and published by the General Board of Health, there is a statement of the number of houses supplied by each of the water companies respectively in each district and subdistrict. The line has not been very accurately drawn where a street, as often happens, is partly in one district and partly in another; and thus, in the recent Report, the subdistricts of St. Saviour's, Southwark, Leather Market, Bermondsey, Battersea, and Peckham, have been represented to contain a few houses supplied by the Lambeth Company, although they do not contain any. With regard to Bermondsey, it is stated in a foot note that some ends of streets may have been included which have passed the registration boundary, and this has happened in other cases; but the errors arising from this cause are limited in amount, and cannot much affect the statistical calculations that I have made. There is also a further imperfection in the account of the water supply of the subdistricts. The numbers which are stated to represent the houses supplied by each water company in each subdistrict are found on adding up the tables not to do so, but to represent the number of houses, minus those situated in streets in which no death occurred; the latter being

* Medical Times and Gazette, Oct. 7, 1854, p. 365.

placed all together at the end of each group of subdistricts which constitutes a district. Streets vary in size from one or two houses to two or three hundred, and the small streets would obviously be the most likely to be exempt from mortality; it could, therefore, do little good to distinguish such streets; however, if thought desirable, this could as well have been done by simply stating the number of the houses, without deducting them from the gross number in each subdistrict. The number of houses in these exempted streets is about one-ninth of the whole. Instead of being able to compare, as I could wish, the mortality in the houses supplied by each company with the exact number of houses supplied, I have only been able to compare it with the number of houses in the streets in which deaths occurred. This will necessarily raise the proportion of deaths about one-ninth; but there is every reason to believe that the relative proportion of deaths in the population supplied by the two companies respectively, which is the real object of the inquiry, will remain almost unaltered.

As the first four weeks of the epidemic did not furnish a sufficient number of cases in all the subdistricts to serve for a statistical inquiry in detail, I have commenced by taking the first seven weeks of the epidemic collectively; and the first of the tables which accompanies this paper exhibits the results of my personal inquiry, when placed in connexion with the number of persons and houses supplied in each subdistrict by each water company respectively.* The reader will observe from the last division of the table that the proportion of deaths was, in every subdistrict, very much greater amongst the population supplied by the Southwark and Vauxhall Company than amongst that supplied by the Lambeth Company, and that the relative mortality is nearly the same throughout, except in two or three instances, where there were but one or two deaths for the basis of calculation amongst the customers of the Lambeth Company. The second table shows the results of that part of the inquiry conducted by Mr. Whiting, treated in a similar manner. In the subdistricts here enumerated, which were supplied, except just on the border of three of them, exclusively by the Southwark and Vauxhall Company, the mortality will be observed to be nearly the same, only a little higher, than

---

* The numbers of deaths in the third division of this Table and the next, are copied from page 85 of the work "On the Mode of Communication of Cholera".

amongst the population supplied by the same company, and mixed with that supplied by the Lambeth Company, as shown in the previous table. In the third table the figures contained in the two first are collected into a more compact form, to show the result of the inquiry during the first part of the epidemic, arranged in districts. The fourth table contains the results of that part of the inquiry made by Dr. Farr, when compared with the population supplied by each water company respectively. It is necessarily arranged arranged in districts—for the results were so published in the Weekly Returns*—and not in subdistricts. The mortality during the last ten weeks of the epidemic was greater than during the first seven weeks, but the reader will observe that a very great disproportion continues in every district between the mortality of the population supplied by one company and that supplied by the other. There is no district to which the supply of both companies extends in which the mortality is not more than three times as great amongst the persons supplied by the Southwark Company as amongst those supplied by the Lambeth Company, and the general result shows a proportion of ninety-one to eighteen, or more than five to one, as was stated before.

In the fifth table the numbers in the previous ones are added together, and fresh calculations made, so as to show the result of the inquiry for the whole epidemic. The instances in which the water supply was not specified, or not ascertained, in the returns made by the district registrars must evidently nearly all have been cases in which the house was supplied by one or other of the water companies, for, if the persons received no such supply, and obtained water from a pump well, canal, or ditch, there could be no difficulty in knowing the fact. Moreover, as the two water companies are guided by precisely the same regulations, the difficulty in ascertaining the supply is exactly the same with regard to one as the other; I, therefore, concluded that I could not be wrong in dividing the non-ascertained cases between the two companies in the same proportion as those which were ascertained, and I have done so at the foot of table v, in order to obtain a complete view of the influence of the water supply during the whole epidemic of 1854. These general results I have employed as the basis of some further calculations.

In table vi I have copied from the Weekly Returns of the

* Loc. cit.

Registrar-General the mortality from cholera in every subdistrict to which the supply of both, or either, of the water companies extends. I have also calculated the number of deaths which would have taken place in each subdistrict according to the number of persons supplied with water by each company respectively, and in accordance with the mortality ascertained for the whole of the population supplied; and it will be observed that the calculated mortality bears a very close relation to the real mortality in each subdistrict. This relation exists with regard both to the gross mortality and to the mortality to each 10,000 living, all through the table, and proves the overwhelming influence which the nature of the water supply exerted over the mortality, overbearing every other circumstance which could be expected to affect the progress of the epidemic. Thus, in the crowded, dirty, and very poor subdistricts of Lambeth Church, first part, and Waterloo, first part, lying by the river side, the mortality was low in consequence of the water supply being chiefly that of the Lambeth Company; whilst in the thinly peopled, and comparatively genteel subdistricts of Clapham and Battersea the mortality was very high, in consequence of the impure water of the Southwark and Vauxhall Company. Taking this inquiry altogether, and considering that the results which were published two years ago, and could only be estimated collectively, are now corroborated in detail through upwards of thirty subdistricts, it probably supplies a greater amount of statistical evidence than was ever brought to bear on a medical subject.

At the latter part of 1854, the General Roard of Healh procured from the two water companies, by order of the Secretary of State, a list of all tbe houses which they supplied, which lists are very valuable, as affording the means of ascertaining the exact water supply of each district and subdistrict separately. By direction of the Scientific Committee of the Board of Health, the lists have been employed in making a supplemental inquiry into the effect of the water supply on cholera. For this purpose they were compared with the lists of deaths at the General Registrar Office, and the results have been embodied in the recent Report of Mr. Simon, previously referred to. There are, however, certain circumstances, which were probably unknown to the Scientific Committee, and which render it impossible that an inquiry, conducted in this manner, could do more than approximate to the truth; and show why it can bear no comparison in point of accuracy to a personal inquiry, made on the spot, at the time of the epidemic. In the first place, throughout the greater part of Lambeth, Newington, and the Borough, the houses are either without numbers, or numbered very irregularly, and the numbers are liable to frequent change, as new houses are built, or older ones re-painted; there are also frequently repetitions of the same number in the same street, and although, in some instances, the companies have returned the names of the occupiers, that can be of no assistance in the case of the poor, who occupy but one or two rooms, and form the greater bulk of the population. In the next place, the poor often furnish, unintentionally, a wrong number to the registrar, even when the houses are regularly numbered. They know their own homes perfectly, but, having no occasion to refer to the number, they partially forget it; and, in the greater number of my personal inquiries, I had to call at two or three houses before I found the one in which the death occurred. For these reasons it follows that, in comparing the lists of the water supply with the lists of deaths, many errors must have occurred; and as the deaths were six times as numerous in the houses supplied by the Southwark and Vauxhall Company as in those supplied by the Lambeth Company, the evident result would be that out of every six mistakes five would transfer a death from the former company to the latter, and only one would transfer a death from the latter company to the former. Another source of error, but operating to a less extent, is, that a number of persons who were attacked with cholera in houses supplied by the Southwark Company died in the workhouses of St. Saviour's, Lambeth, and Newington, which were supplied by the Lambeth Compan. It need excite no surprise, therefore, that the supplemental inquiry, embodied in the recent Report, instead of showing a mortality of 160 and 27 for the population supplied by the two water companies, or a difference of 6 to 1, showed a mortality of 125 and 37 per 10,000, or a difference of only 3½ to 1. It must be obvious, however, independently of the above facts, that a difference of three and a-half to one would not explain the great difference in the mortality of the various districts and subdistricts. The epidemic of 1853 is included with that of 1854 in Mr. Simon's Report; but as there were but few deaths in 1853, and those chiefly amongst the population supplied by the Southwark Company, this circumstance would not much affect his results.

It is probable that, when the facts brought to light by this inquiry are sufficientiy known, no one will deny the influence of impure water in promoting the mortality of cholera; but it must not be supposed that it is mere impurity of an ordinary kind that causes the disease, for there are innumerable facts to prove that ordinary impurities have no such effect, and that it is only when the specific morbid matter of the disease gains access to the water that cholera is propagated. Thousands of people drank water from their own neglected cisterns, during the late epidemic, as impure as that of the Southwark and Vauxhall Company, without ill effect. An inquiry made by the vestry of St. James', Westminster, proved that the contents of a cesspool had been percolating for months through the three feet of earth which separated it from the pump well in Broad Street; but although hundreds of people were daily drinking the water, and cholera was extending fearfully in many parts of London, only a few scattered cases occurred in the streets near the pump till the end of August, when, a case having happened amongst the persons using the privy connected with the cesspool above mentioned, more than five hundred persons were attacked within two or three days.

In the cases in which the cholera poison gains access to a limited supply of drinking water, such as a tank or pump-well, the outbreak it occasions is always sudden, violent, and limited; but when a river is the medium of the propagation of the disease, its progress is more gradual and extended, being diffused amongst the whole population using the water.

It is hardly necessary to remark, that every circumstance which proves the communication of cholera through the medium of water, corroborates the views, explained at the beginning of this paper, regarding its propagation in the crowded houses of the poor; for it cannot be supposed that a morbid matter, which can produce its specific effects after being diffused and distributed through a quantity of water, could fail to act in an undiluted state.

It was my intention to make some remarks on the drainage and water supply of towns, but this communication has already exceeded the limits which I prescribed for it.

Sackville Street.

TABLE I. *Shewing the results of the Author's personal Inquiry in Twenty-One Sub-Districts.*

| Registration Districts. | Registration Sub-Districts. | Number of inhabited houses in 1851. | Population in 1851. | Estimated constant population per house. | "Number of houses, and estimated number of persons, supplied in 1854 with water as under." By Southwark and Vauxhall Co. No. of houses. | By Southwark and Vauxhall Co. Estim. Population. | By the Lambeth Company. No. of houses. | By the Lambeth Company. Estim. Population. | Water supply of the houses in which fatal attacks of cholera took place during first seven weeks of epidemic of 1854. Southwark and Vauxhall Co. | Lambeth Co. | Thames, canals, or ditches. | From pump-wells. | Supply not ascertained. | Deaths from cholera in first 7 weeks of epidemic of 1854. | Mortality per 10,000 supplied with water as under. Southwark and Vauxhall Co. | Lambeth Co. |
|---|---|---|---|---|---|---|---|---|---|---|---|---|---|---|---|---|
| St. Saviour, Southw. | 1. Christchurch ........ | 1,887 | 16,022 | 8·5 | 343 | 2,915 | 1,557 | 13,234 | 11 | 13 | 0 | 0 | 1 | 25 | 37·7 | 9·9 |
| St. George, Southw. | 1. Kent Road .......... | 2,558 | 18,126 | 7·1 | 1,779 | 12,630 | 563 | 3,997 | 52 | 5 | 0 | 0 | 0 | 57 | 41·1 | 12·5 |
| | 2. Borough Road ...... | 2,069 | 15,862 | 7·7 | 1,176 | 8,937 | 878 | 6,672 | 61 | 7 | 0 | 0 | 3 | 71 | 68·2 | 10·4 |
| | 3. London Road........ | 2,365 | 17,836 | 7·5 | 383 | 2,872 | 1,533 | 11,497 | 21 | 8 | 0 | 0 | 0 | 29 | 73·1 | 6·9 |
| Newington ........ | 1. Trinity ............ | 3,224 | 20,922 | 6·5 | 1,661 | 10,132 | 1,372 | 8,370 | 52 | 6 | 0 | 0 | 0 | 58 | 51·3 | 7·1 |
| | 2. St. Peter, Walworth .. | 4,925 | 29,861 | 6·1 | 2,340 | 14,274 | 1,758 | 10,724 | 84 | 4 | 0 | 0 | 2 | 90 | 58·8 | 3·7 |
| | 3. St. Mary............ | 2,309 | 14,033 | 6·1 | 489 | 2,983 | 899 | 5,484 | 19 | 1 | 0 | 1 | 0 | 21 | 64·5 | 1·8 |
| Lambeth.......... | 1. Waterloo, part 1 .... | 1,729 | 14,088 | 8·1 | 438 | 3,548 | 1,474 | 11,939 | 9 | 1 | 0 | 0 | 0 | 10 | 25·6 | 0·8 |
| | 2. Waterloo, part 2 .... | 2,191 | 18,348 | 8·4 | 864 | 7,171 | 1,510 | 12,533 | 25 | 8 | 2 | 1 | 0 | 36 | 34·8 | 6·3 |
| | 3. Lambeth church, pt. 1 | 2,451 | 18,409 | 7·5 | 415 | 3,113 | 2,117 | 15,878 | 6 | 9 | 1 | 0 | 2 | 18 | 19·2 | 5·6 |
| | 4. Lambeth church, pt. 2 | 3,849 | 26,784 | 7·0 | 1,124 | 7,868 | 2,289 | 16,023 | 34 | 13 | 0 | 1 | 5 | 53 | 42·9 | 8·1 |
| | 5. Kennington, part 1 .. | 3,977 | 24,261 | 6·1 | 2,586 | 15,775 | 444 | 2,708 | 63 | 5 | 0 | 3 | 0 | 71 | 39·9 | 18·4 |
| | 6. Kennington, part 2 .. | 3,288 | 18,848 | 5·7 | 1,206 | 7,874 | 986 | 5,620 | 34 | 3 | 0 | 1 | 0 | 38 | 43·2 | 5·7 |
| | 7. Brixton ............ | 2,362 | 14,610 | 6·1 | 310 | 1,922 | 1,509 | 9,356 | 5 | 2 | 0 | 0 | 2 | 9 | 26·0 | 2·1 |
| | 8. Norwood............ | 600 | 3,977 | 6·6 | 0 | 0 | 160 | 1,066 | 0 | 2 | 5 | 1 | 0 | 8 | .. | 18·7 |
| Wandsworth ...... | 3. Wandsworth ........ | 1,522 | 9,611 | 6·3 | 144 | 907 | 15 | 94 | 1 | 0 | 8 | 2 | 0 | 11 | 11·0 | .. |
| | 4. Putney ............ | 918 | 5,280 | 5·7 | 13 | 74 | 0 | 0 | 0 | 0 | 0 | 1 | 0 | 1 | .. | .. |
| | 5. Streatham .......... | 1,419 | 9,023 | 6·4 | 0 | 0 | 515 | 3,244 | 0 | 1 | 0 | 5 | 0 | 6 | .. | 3·0 |
| Camberwell ...... | 1. Dulwich ............ | 259 | 1,632 | 6·3 | 0 | 0 | 4 | 25 | 0 | 0 | 0 | 0 | 0 | 0 | .. | .. |
| | 4. St. George .......... | 2,845 | 15,849 | 5·6 | 767 | 4,295 | 971 | 5,437 | 30 | 9 | 0 | 2 | 1 | 42 | 69·8 | 16·5 |
| Lewisham ........ | 5. Sydenham .......... | 801 | 4,501 | 5·6 | 0 | 0 | unkno. | unkno. | 0 | 1 | 0 | 2 | 1 | 4 | .. | .. |
| Totals ...................... | | 47,548 | 317,883 | 6·6 | 16,038 | 107,290 | 20,554 | 143,901 | 507 | 98 | 16 | 20 | 17 | 658 | 47·2 | 6·8 |

### Table II.

*Shewing the results of the Inquiry made by Mr. Whiting in Eleven Sub-Districts.*

| Registration Districts. | Registration Sub-Districts. | Number of inhabited houses in 1851. | Population in 1851. | Estimated constant population per house. | "Number of houses, and estimated number of persons, supplied in 1854 with water as under." By Southwark and Vauxhall Co. No. of houses. | By Southwark and Vauxhall Co. Estim. Population. | By the Lambeth Company. No. of houses. | By the Lambeth Company. Estim. Population. | Water supply of the houses in which fatal attacks of cholera took place during first seven weeks of epidemic of 1854. Southwark and Vauxhall Co. | Lambeth Co. | Thames, canals, or ditches. | Pump-wells. | Supply not ascertained. | Deaths from cholera in first 7 weeks of epidemic of 1854. | Mortality per 10,000 supplied with water as under. Southwark and Vauxhall Co. | Lambeth Co. |
|---|---|---|---|---|---|---|---|---|---|---|---|---|---|---|---|---|
| St. Saviour, Southw. | 2. St. Saviour......... | 2,713 | 19,709 | 7·3 | 2,238 | 16,337 | 123 | 898 | 115 | 0 | 10 | 0 | 0 | 125 | 70·3 | .. |
| St. Olave, Southwark | 1. St. Olave............ | 880 | 8,015 | 9·1 | 961 | 8,745 | 0 | 0 | 43 | 0 | 5 | 0 | 5 | 53 | 49·1 | .. |
| | 2. St. John, Horselydown | 1,480 | 11,360 | 7·7 | 1,170 | 9,360 | 0 | 0 | 48 | 0 | 3 | 0 | 0 | 51 | 50·1 | .. |
| Bermondsey ...... | 1. St. James .......... | 2,863 | 18,899 | 6·6 | 3,511 | 23,173 | 105 | 693 | 102 | 0 | 21 | 0 | 0 | 123 | 44·0 | .. |
| | 2. St. Mary Magdalen .. | 1,865 | 13,934 | 7·5 | 2,301 | 17,258 | 0 | 0 | 83 | 0 | 4 | 0 | 0 | 87 | 48·0 | .. |
| | 3. Leather Market...... | 2,279 | 15,295 | 6·7 | 2,090 | 14,003 | 163 | 1,092 | 81 | 0 | 0 | 0 | 0 | 81 | 57·8 | .. |
| Wandsworth ...... | 1. Clapham............. | 2,657 | 16,290 | 6·1 | 1,106 | 6,747 | 22 | 134 | 19 | 0 | 0 | 5 | 0 | 24 | 28·1 | .. |
| | 2. Battersea .......... | 1,760 | 10,560 | 6·0 | 1,046 | 6,276 | 46 | 276 | 42 | 0 | 8 | 4 | 0 | 54 | 66·9 | .. |
| Camberwell ...... | 2. Camberwell ....... | 2,851 | 17,742 | 6·2 | 1,474 | 9,139 | 103 | 639 | 96 | 0 | 0 | 0 | 0 | 96 | 104·8 | .. |
| | 3. Peckham .......... | 3,457 | 19,444 | 5·6 | 971 | 5,438 | 70 | 392 | 59 | 0 | 0 | 0 | 0 | 59 | 108·4 | .. |
| Rotherhithe ...... | Rotherhithe ........ | 2,792 | 17,805 | 6·4 | 1,909 | 12,218 | 0 | 0 | 68 | 0 | 35 | 0 | 0 | 103 | 55·6 | .. |
| Totals ....................... | | 25,597 | 169,053 | 6·5 | 18,777 | 128,694 | 632 | 4,124 | 756 | 0 | 86 | 9 | 5 | 856 | 58·7 | .. |
| Totals of Table I. ............... | | 47,548 | 317,883 | 6·6 | 16,038 | 107,290 | 20,554 | 143,901 | 507 | 98 | 16 | 20 | 17 | 658 | 47·2 | 6·8 |
| Houses in streets where no death occurred.... | | .. | .. | 6·4 | 4,500 | 28,929 | 3,643 | 23,338 | 0 | 0 | 0 | 0 | 0 | 0 | .. | .. |
| Not identified .......................... | | .. | .. | 6·6 | 411 | 2,712 | 25 | 165 | .. | .. | .. | .. | .. | .. | .. | .. |
| Totals of thirty-two Sub-districts . | | 73,145 | 486,936 | 6·6 | 39,726 | 267,625 | 24,854 | 171,528 | 1263 | 98 | 102 | 29 | 22 | 1514 | 47·2 | 5·7 |

### Table III.

*Shewing the results of the whole Inquiry during the First Seven Weeks of the Epidemic, arranged in Districts.*

| Registration Districts. | Number of inhabited houses in 1851. | Population in 1851. | Estimated constant population per house. | "Number of houses, and estimated number of persons, supplied in 1854 with water as under." By the Southwark and Vauxhall Co. | | By the Lambeth Company. | | Water supply of the houses in which fatal attacks of cholera took place during first seven weeks of epidemic of 1854. | | | | | Deaths in first seven weeks of epidemic. | Mortality per 10,000 supplied with water as under. | |
|---|---|---|---|---|---|---|---|---|---|---|---|---|---|---|---|
| | | | | No. of houses. | Estimated population | No. of houses. | Estimated population | Southwark and Vauxhall Co. | Lambeth Co. | Thames, canals, or ditches. | Pump-wells. | Supply not ascertained. | | Southwark and Vauxhall Co. | Lambeth Co. |
| St. Saviour, Southwark ...... | 4,600 | 35,731 | 7·8 | 2,631 | 19,617 | 1,689 | 14,201 | 126 | 13 | 10 | 0 | 1 | 150 | 64·4 | 9·1 |
| St. Olave, Southwark ........ | 2,360 | 19,375 | 8·2 | 2,193 | 18,638 | 0 | 0 | 91 | 0 | 8 | 0 | 5 | 104 | 48·8 | .. |
| Bermondsey................ | 7,007 | 48,128 | 6·9 | 8,402 | 57,884 | 268 | 1,785 | 266 | 0 | 25 | 0 | 0 | 291 | 45·9 | .. |
| St. George, Southwark ...... | 6,992 | 51,824 | 7·4 | 3,419 | 25,039 | 3,183 | 23,712 | 134 | 20 | 0 | 0 | 3 | 157 | 53·5 | 8·4 |
| Newington ................ | 10,458 | 64,816 | 6·2 | 5,224 | 31,940 | 5,473 | 33,531 | 155 | 11 | 0 | 1 | 2 | 169 | 48·5 | 3·2 |
| Lambeth .................. | 20,447 | 139,325 | 6·8 | 8,077 | 54,982 | 11,763 | 83,786 | 176 | 43 | 8 | 7 | 9 | 243 | 32·8 | 4·9 |
| Wandsworth ............... | 8,276 | 50,764 | 6·1 | 3,028 | 18,390 | 618 | 3,870 | 62 | 1 | 16 | 17 | 0 | 96 | 33·7 | 3·3 |
| Camberwell ............... | 9,412 | 54,667 | 5·8 | 4,005 | 23,472 | 1,835 | 10,478 | 185 | 9 | 0 | 2 | 1 | 197 | 78·8 | 8·5 |
| Rotherhithe............... | 2,792 | 17,805 | 6·4 | 2,336 | 14,951 | 0 | 0 | 68 | 0 | 35 | 0 | 0 | 103 | 45·4 | .. |
| Sub-district of Sydenham.... | 801 | 4,501 | 5·6 | 0 | 0 | unknow. | unknow. | 0 | 1 | 0 | 2 | 1 | 4 | .. | .. |
| Not identified ............. | .. | .. | 6·6 | 411 | 2,712 | 25 | 165 | .. | .. | .. | .. | .. | .. | .. | .. |
| Totals............... | 73,145 | 486,936 | 6·7 | 39,726 | 267,625 | 24,854 | 171,528 | 1263 | 98 | 102 | 29 | 22 | 1514 | 47·2 | 5·7 |

### Table IV.

*The Inquiry of the General Register Office during the Last Ten Weeks of the Epidemic.*

| Registration District. | Number of inhabited houses in 1851. | Population in 1851. | Estimated constant population per house. | " Number of houses, and estimated number of persons, supplied in 1854 with water as under." By the Southwark and Vauxhall Co. | | By the Lambeth Company. | | Water supply of the houses in which fatal attacks of cholera took place. | | | | Deaths from cholera in last ten weeks of epidemic. | Mortality per 10,000 supplied with water as under. | |
|---|---|---|---|---|---|---|---|---|---|---|---|---|---|---|
| | | | | No. of houses. | Estimated population | No. of houses. | Estimated population | Southwark and Vauxhall Co. | Lambeth Co. | Pump-wells and other sources. | Supply not ascertained. | | Southwark and Vauxhall Co. | Lambeth Co. |
| St. Saviour, Southwark ...... | 4,600 | 35,721 | 7·8 | 2,631 | 19,617 | 1,689 | 14,201 | 280 | 59 | 0 | 2 | 341 | 143 | 41 |
| St. Olave, Southwark ........ | 2,360 | 19,375 | 8·2 | 2,193 | 18,638 | 0 | 0 | 186 | 0 | 0 | 23 | 209 | 100 | .. |
| Bermondsey .............. | 7,007 | 48,128 | 6·9 | 8,402 | 57,884 | 268 | 1,785 | 555 | 0 | 0 | 0 | 555 | 96 | .. |
| St. George, Southwark ...... | 6,992 | 51,824 | 7·4 | 3,419 | 25,039 | 3,183 | 23,712 | 254 | 79 | 0 | 53 | 386 | 101 | 33 |
| Newington ............... | 10,458 | 64,816 | 6·2 | 5,224 | 31,940 | 5,473 | 33,531 | 303 | 47 | 1 | 174 | 525 | 95 | 14 |
| Lambeth .................. | 20,447 | 139,325 | 6·8 | 8,077 | 54,982 | 11,763 | 83,786 | 349 | 95 | 9 | 231 | 684 | 63 | 11 |
| Wandsworth .............. | 8,276 | 50,764 | 6·1 | 3,028 | 18,390 | 618 | 3,870 | 206 | 6 | 73 | 40 | 325 | 112 | 15 |
| Camberwell ................ | 9,412 | 54,667 | 5·8 | 4,005 | 23,472 | 1,835 | 10,478 | 167 | 24 | 113 | 48 | 352 | 71 | 23 |
| Rotherhithe................ | 2,792 | 17,805 | 6·4 | 2,336 | 14,951 | 0 | 0 | 139 | 0 | 11 | 30 | 180 | 93 | .. |
| Greenwich&sub-dis.Sydenham | .. | .. | .. | .. | .. | .. | .. | 4 | 3 | .. | .. | 7 | .. | .. |
| Houses not identified........ | .. | .. | 6·6 | 411 | 2,712 | 25 | 165 | .. | .. | .. | .. | .. | .. | .. |
| Totals .............. | 72,344 | 482,435 | 6·7 | 39,726 | 267,625 | 24,854 | 171,528 | 2,443 | 313 | 207 | 601 | 3,564 | 91 | 18 |

### Table V.

*Shewing the results of the Inquiry for the whole Epidemic of* 1854.

| Registration Districts. | Number of inhabited houses in 1851. | Population in 1851. | Estimated constant population per house. | "Number of houses, and estimated number of persons, supplied in 1854 with water as under." By the Southwark and Vauxhall Co. | | By the Lambeth Company. | | Water supply of the houses in which fatal attacks of cholera took place. | | | | Deaths from cholera in the epidemic of 1854. | Mortality per 10,000 supplied with water as under. | |
|---|---|---|---|---|---|---|---|---|---|---|---|---|---|---|
| | | | | No. of houses. | Estimated population | No, of houses. | Estimated population | Southwark and Vauxhall Co. | Lambeth Co. | Pump-wells and other sources. | Supply not ascertained. | | Southwark and Vauxhall Co. | Lambeth Co. |
| St. Saviour, Southwark ...... | 4,600 | 35,731 | 7·8 | 2,631 | 19,617 | 1,689 | 14,201 | 406 | 72 | 10 | 3 | 491 | 207 | 50 |
| St. Olave, Southwark ........ | 2,360 | 19,375 | 8·2 | 2,193 | 18,638 | 0 | 0 | 277 | 0 | 8 | 28 | 313 | 148 | .. |
| Bermondsey .............. | 7,007 | 48,128 | 6·9 | 8,402 | 57,884 | 268 | 1,785 | 821 | 0 | 25 | 0 | 846 | 142 | .. |
| St. George, Southwark ...... | 6,992 | 51,824 | 7·4 | 3,419 | 25,039 | 3,183 | 23,712 | 388 | 99 | 0 | 56 | 543 | 155 | 41 |
| Newington ................ | 10,458 | 64,816 | 6·2 | 5,224 | 31,940 | 5,473 | 33,531 | 458 | 58 | 2 | 176 | 694 | 143 | 17 |
| Lambeth ................. | 20,447 | 139,325 | 6·8 | 8,077 | 54,982 | 11,763 | 83,786 | 525 | 138 | 24 | 240 | 927 | 96 | 16 |
| Wandsworth .............. | 8,276 | 50,764 | 6·1 | 3,028 | 18,390 | 618 | 3,870 | 268 | 7 | 106 | 40 | 421 | 145 | 18 |
| Camberwell ............... | 9,412 | 54,607 | 5·8 | 4,005 | 23,472 | 1,835 | 10,478 | 352 | 33 | 115 | 49 | 549 | 150 | 31 |
| Rotherhithe............... | 2,792 | 17,805 | 6·4 | 2,336 | 14,951 | 0 | 0 | 207 | 0 | 46 | 30 | 283 | 138 | .. |
| Greenwich & sub-dis. Sydenham | .. | .. | .. | .. | .. | .. | .. | 4 | 4 | 2 | 1 | 11 | .. | .. |
| Houses not identified........ | .. | .. | 6·6 | 411 | 2,712 | 25 | 165 | .. | .. | .. | .. | .. | .. | .. |
| Totals .............. | 72,344 | 482,435 | 6·7 | 39,726 | 267,625 | 24,854 | 171,528 | 3,706 | 411 | 338 | 623 | 5,078 | 138 | 23 |
| Non-ascertained cases distributed in proportion of others | .. | .. | .. | .. | .. | .. | .. | 561 | 62 | .. | .. | .. | .. | .. |
| Population (Registrar-General) | .. | .. | .. | .. | 266,516 | .. | 173,748 | 4,267 | 473 | 338 | .. | 5,078 | 160 | 27 |

### Table VI.

*The Mortality from Cholera in* 1854, *in Thirty-one Sub-Districts, as compared with Calculations founded on the Results shewn in* Table v.

| Registration Districts. | Registration Sub-Districts. | Population in 1851. | Estimated population supplied with water as under. | | | Deaths from cholera in 1854. | | Calculated mortality in the population, supplied with water as under. | | | |
|---|---|---|---|---|---|---|---|---|---|---|---|
| | | | Southwark and Vauxhall Co. | Lambeth Co. | Both Companies together. | Total deaths. | Deaths per 10,000 living. | Southwark and Vauxhall Co. at 160 per 10,000. | Lambeth Co. at 27 per 10,000. | The two Companies. | Calculated deaths per 10,000 supplied by the two Companies. |
| St. Saviour, Southw. - | 1. Christchurch - - - - | 16,022 | 2,915 | 13,234 | 16,149 | 113 | 71 | 46 | 36 | 82 | 57 |
| | 2. St. Saviour - - - - | 19,709 | 16,337 | 898 | 17,235 | 378 | 192 | 261 | 2 | 263 | 153 |
| St. Olave - - - - | 1. St. Olave - - - - - | 8,015 | 8,745 | 0 | 8,745 | 161 | 201 | 140 | 0 | 140 | 160 |
| | 2. St. John, Horselydown | 11,360 | 9,360 | 0 | 9,360 | 152 | 134 | 150 | 0 | 150 | 160 |
| Bermondsey - - - | 1. St. James - - - - - | 18,899 | 23,173 | 693 | 23,866 | 362 | 192 | 370 | 2 | 372 | 156 |
| | 2. St. Mary Magdalen - | 13,934 | 17,258 | 0 | 17,258 | 247 | 177 | 276 | 0 | 276 | 160 |
| | 3. Leather Market - - - | 15,295 | 14,003 | 1,092 | 15,095 | 237 | 155 | 224 | 3 | 227 | 150 |
| St. George, Southw. - | 1. Kent Road - - - - | 18,126 | 12,630 | 3,997 | 16,627 | 177 | 98 | 202 | 11 | 213 | 134 |
| | 2. Borough Road - - - | 15,862 | 8,937 | 6,672 | 15,609 | 271 | 171 | 143 | 18 | 161 | 104 |
| | 3. London Road - - - | 17,836 | 2,872 | 11,497 | 14,369 | 95 | 53 | 46 | 31 | 79 | 55 |
| Newington - - - - | 1. Trinity - - - - - | 20,922 | 10,132 | 8,370 | 18,502 | 211 | 101 | 162 | 22 | 184 | 99 |
| | 2. St. Peter, Walworth - | 29,861 | 14,274 | 10,724 | 24,998 | 391 | 131 | 228 | 29 | 257 | 103 |
| | 3. St. Mary - - - - - | 14,033 | 2,983 | 5,484 | 8,467 | 92 | 66 | 48 | 15 | 63 | 74 |

| | | | | | | | | | | | |
|---|---|---|---|---|---|---|---|---|---|---|---|
| Lambeth | 1. Waterloo, part 1 | 14,088 | 3,548 | 11,939 | 15,487 | 59 | 42 | 57 | 31 | 86 | 55 |
| | 2. Waterloo, part 2 | 18,348 | 7,171 | 12,533 | 19,704 | 118 | 64 | 115 | 34 | 149 | 76 |
| | 3. Lambeth church, pt. 1 | 18,409 | 3,113 | 15,878 | 18,991 | 49 | 27 | 50 | 43 | 93 | 49 |
| | 4. Lambeth church, pt. 2 | 26,784 | 7,868 | 16,023 | 23,891 | 195 | 73 | 126 | 43 | 167 | 71 |
| | 5. Kennington, part 1 | 24,261 | 15,775 | 2,708 | 18,483 | 305 | 126 | 253 | 7 | 260 | 146 |
| | 6. Kennington, part 2 | 18,848 | 7,874 | 5,620 | 13,494 | 143 | 75 | 126 | 15 | 141 | 105 |
| | 7. Brixton | 14,610 | 1,922 | 9,356 | 11,278 | 48 | 33 | 31 | 25 | 56 | 49 |
| | 8. Norwood | 3,977 | 0 | 1,066 | 1,066 | 10 | 25 | 0 | 3 | 3 | 28 |
| Wandsworth | 1. Clapham | 16,290 | 6,747 | 134 | 6,881 | 167 | 103 | 108 | 0 | 108 | 158 |
| | 2. Battersea | 10,560 | 6,276 | 276 | 6,552 | 171 | 162 | 100 | 1 | 101 | 152 |
| | 3. Wandsworth | 9,611 | 907 | 94 | 1,001 | 59 | 61 | 15 | 0 | 15 | 149 |
| | 4. Putney | 5,280 | 74 | 0 | 74 | 9 | 17 | 1 | 0 | 1 | 160 |
| | 5. Streatham | 9,023 | 0 | 3,244 | 3,244 | 15 | 17 | 0 | 9 | 9 | 27 |
| Camberwell | 1. Dulwich | 1,632 | 0 | 25 | 25 | 0 | 0 | 0 | 0 | 0 | 0 |
| | 2. Camberwell | 17,742 | 9,139 | 639 | 9,778 | 242 | 136 | 146 | 2 | 148 | 151 |
| | 3. Peckham | 19,444 | 5,438 | 392 | 5,830 | 175 | 90 | 87 | 1 | 88 | 151 |
| | 4. St. George | 15,849 | 4,295 | 5,437 | 9,732 | 132 | 83 | 69 | 15 | 84 | 86 |
| Rotherhithe | Rotherhithe | 17,805 | 12,218 | 0 | 12,218 | 283 | 159 | 196 | 0 | 196 | 160 |
| Houses supplied in streets where no death occurred | | .. | 28,929 | 23,338 | 52,267 | .. | .. | .. | .. | .. | .. |
| Houses not identified | | .. | 2,712 | 165 | 2,877 | .. | .. | .. | .. | .. | .. |
| Totals | | 482,435 | 267,625 | 171,528 | 439,153 | 5,067 | 105 | 4,282 | 462 | 4,744 | 108 |
| Population as estimated by the Registrar-General | | .. | 266,516 | 173,748 | 440,264 | .. | .. | 4,267 | 473 | 4,740 | 108 |

